# Public knowledge, attitudes, and perceptions of the use of ChatGPT for self-diagnosis, health information, and medication use: a cross-sectional study

**DOI:** 10.3389/fpubh.2026.1936664

**Published:** 2026-10-16

**Authors:** Mohammad S. Alzahrani, Daniyah A. Almarghalani, Alaa M. Hammad, Adnan Alharbi, Manar khaled Alhayfani, Mohammed S. Alharthi, Tahani Momenah, Khulood A. Almehmadi, Amal H. Alzahrani, Daniyah Mansour Alazwari, Ahdab Safar Althobaiti, Mjd Awadh Alkhudaidi, Ekram Saad Aljudayi, Dimah Zuwayyid Alsufyani, Hawazin Saleh Alsufyani

**Affiliations:** 1 Department of Clinical Pharmacy, College of Pharmacy, Taif University, Taif, Saudi Arabia; 2 Department of Pharmacology and Toxicology, College of Pharmacy, Taif University, Taif, Saudi Arabia; 3 Stroke Research Unit, Taif University, Taif, Saudi Arabia; 4 Department of Pharmacy, Faculty of Pharmacy, Al-Zaytoonah University of Jordan, Amman, Jordan; 5 Pharmaceutical Practices Department, College of Pharmacy, Umm Al-Qura University, Makkah, Saudi Arabia; 6 Department of Pharmacology and Toxicology, Faculty of Pharmacy, King Abdulaziz University, Jeddah, Saudi Arabia

**Keywords:** artificial intelligence, ChatGPT, digital health literacy, health information, risk perception, self-diagnosis

## Abstract

**Background:**

The increasing availability of generative artificial intelligence (AI) tools, such as ChatGPT, has changed the ways individuals access health information. While these technologies offer rapid, convenient access to information, concerns remain about their accuracy, safety, and potential influence on health-related decision-making. Understanding public knowledge, attitudes, and perceptions toward ChatGPT is essential to support its safe and responsible use in healthcare contexts.

**Objective:**

To assess the use of ChatGPT for health-related purposes and examine demographic, knowledge, attitude, perception, and risk–reward appraisal factors associated with its use among adults recruited from the Western Region of Saudi Arabia.

**Methods:**

In this cross-sectional study, 735 participants completed an online questionnaire. The instrument selected constructs from the unified theory of acceptance and use of technology (UTAUT) and was informed by the technology acceptance model (TAM), which captures demographics, health literacy, ChatGPT use, and multi-item Likert scales measuring knowledge, attitudes, perceptions, and risk–reward appraisal. We applied descriptive statistics, chi-square tests, t tests, ANOVA, and multivariable binary logistic regression.

**Results:**

Overall, 66.3% of the participants reported using ChatGPT for health-related purposes. The participants demonstrated good awareness of ChatGPT’s limitations, particularly the importance of consulting healthcare professionals for accurate diagnosis and safe medication use (mean = 4.58 ± 0.74). Attitudes toward ChatGPT were moderate, whereas perceptions of its usefulness and accessibility were generally positive. Compared with nonusers, ChatGPT users presented significantly higher attitudes, perceptions, and risk–reward appraisal scores (all *p* < 0.01), whereas knowledge scores were comparable between the two groups. In the multivariable logistic regression analysis, compared with participants aged ≥45 years, those aged 18–24 years (aOR = 5.37, 95% CI: 2.34–12.34, *p* < 0.001) and 25–44 years (aOR = 2.28, 95% CI: 1.32–3.96, *p* = 0.003) had higher odds of reported use. Participants with a bachelor’s degree (aOR = 1.82, 95% CI: 1.15–2.89, *p* = 0.011) or postgraduate education (aOR = 2.80, 95% CI: 1.34–5.85, *p* = 0.006), as well as students (aOR = 2.56, 95% CI: 1.29–5.09, *p* = 0.007), also had higher odds of use. Higher total perception scores were independently associated with reported ChatGPT use (aOR = 1.19, 95% CI: 1.12–1.26, *p* < 0.001).

**Conclusion:**

ChatGPT is widely used for health-related purposes, particularly among younger and more educated individuals. Despite good awareness of its limitations, more favorable perceptions were independently associated with reported use, emphasizing the need for digital health literacy and guidance for safe AI use in healthcare.

## Graphical abstract

**Figure:**
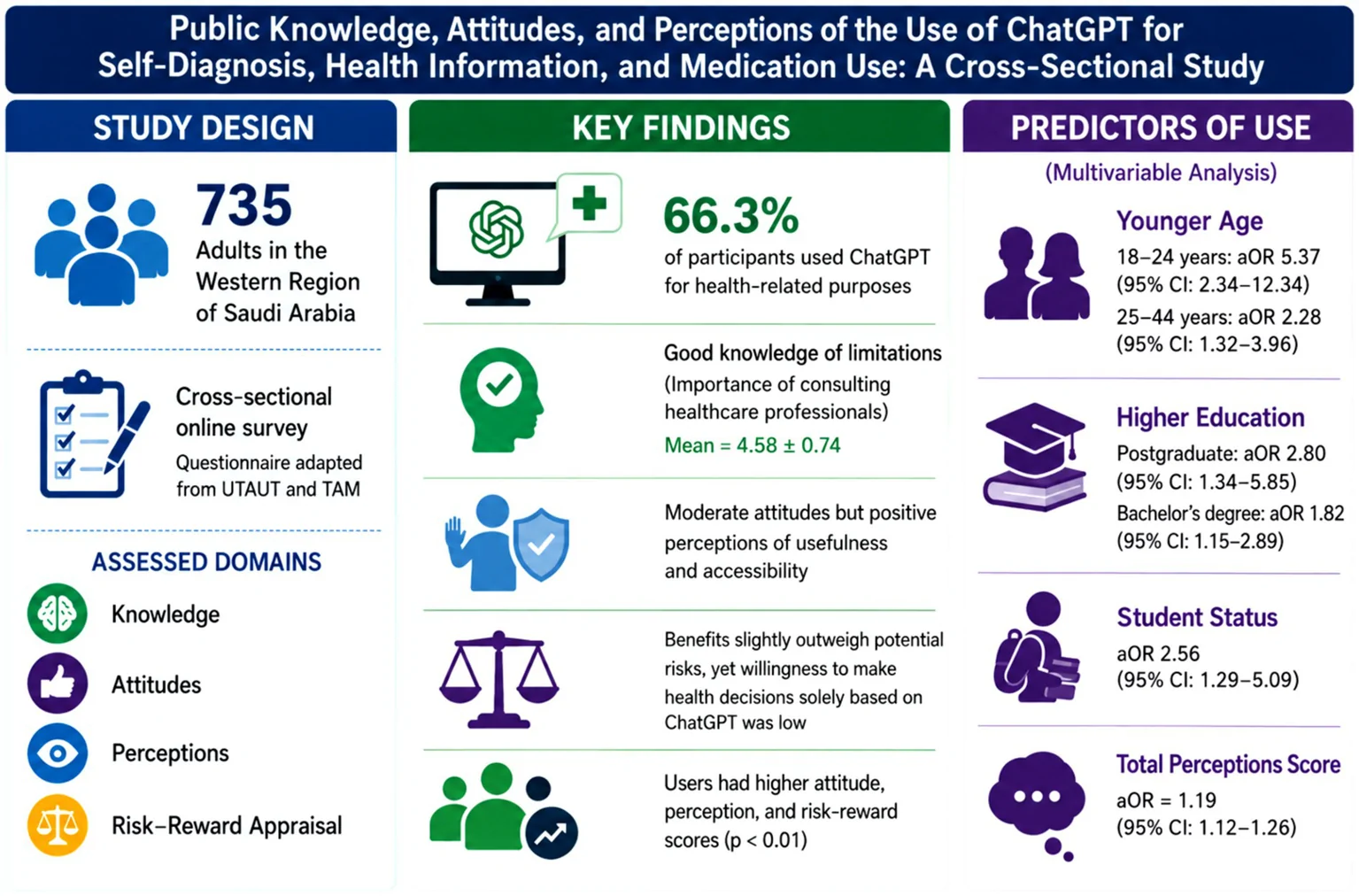
Overview of the study design and principal findings. A cross-sectional online survey of 735 adults from the western region of Saudi Arabia assessed knowledge, attitudes, perceptions, and risk–reward appraisals regarding the use of ChatGPT for health-related purposes. Overall, 66.3% of the participants reported using ChatGPT for health-related purposes. Younger age, higher educational level, student status and total perceptions score were independently associated with ChatGPT use, while participants demonstrated good awareness of its limitations despite generally positive perceptions of its usefulness.

## 1 Introduction

Artificial intelligence (AI) has rapidly become embedded in everyday life, with large language models (LLMs), such as ChatGPT, transforming how individuals access and interact with information. Since its public release in late 2022, ChatGPT has been widely used for educational, professional, economic, writing, research, and scientific publications and, more recently, health-related applications (1–4). Unlike traditional search engines, ChatGPT offers conversational responses that simplify complex information into an easily understandable format and allow users to ask follow-up questions and receive immediate feedback (5, 6). This has led many people to increasingly use generative AI to learn about symptoms, diseases, medications, lab results, and preventive health measures before meeting with healthcare professionals (7–9).

As the use of ChatGPT for health information increases, both benefits and challenges arise. It offers quick access to medical information, potentially improving health literacy and helping people understand medical jargon or prepare for doctor visits. However, ChatGPT was not designed to be a clinical decision-support system and might produce incorrect, incomplete, or fabricated information, often referred to as AI “hallucinations” (10, 11). ChatGPT has potential as a supportive and helpful tool for patient education and general medical guidance; however, its performance remains inconsistent across different clinical scenarios and is less reliable for individualized care, understanding guidelines, and managing complications. Consequently, it should be used to complement, not replace, professional clinical judgment, with further research needed to define its optimal role in healthcare (12, 13).

Among the most common health uses of ChatGPT are self-diagnosis and medication information. While these functions can enhance access to health information, excessive reliance on AI-generated advice may lead to delays in seeking medical attention, misdiagnosis, incorrect medication use, or increased health anxiety (14, 15). Public acceptance of ChatGPT in healthcare is shaped by its perceived usefulness, trust, and emotional engagement with conversational AI. Despite awareness of its limitations, many users continue to perceive AI-generated responses as credible and authoritative, suggesting a disconnect between awareness and actual reliance. Moreover, perceived risk remains an important factor influencing the willingness to use ChatGPT for health-related purposes. Understanding these perceptions is critical for informing the safe, responsible, and effective integration of AI-assisted technologies into healthcare (16–18).

These patterns can be interpreted using established theories of technology adoption. The technology acceptance model (TAM) proposes that perceived usefulness and perceived ease of use are the primary determinants of technology adoption (19), whereas the unified theory of acceptance and use of technology (UTAUT) further incorporates factors such as social influence and facilitating conditions to explain behavioral intention and actual technology use (20). In the context of generative AI, these frameworks suggest that individuals may continue to use ChatGPT despite recognizing its limitations because of its convenience and perceived utility. Examining public knowledge, attitudes, and perceptions is therefore important for understanding the factors that are associated with adoption/use of AI-assisted health information tools.

Although the opportunities and challenges associated with ChatGPT are global, their adoption and impact are likely to vary across healthcare systems, regulatory environments, and sociocultural contexts (21, 22). Saudi Arabia provides a particularly relevant setting in which to examine these issues. As part of Saudi Vision 2030, the country has invested substantially in digital health transformation, supported by widespread internet access, high smartphone penetration, and a digitally engaged population, through the initiation of the Electronic Health Records and telemedicine services initiative (23). Recent evidence suggests that the Saudi public is increasingly using ChatGPT for health-related purposes. In a national cross-sectional study, Alhomoud et al. reported that the Saudi public increasingly relies on ChatGPT for medication-related information. Despite valuing its convenience and rapid response, many participants expressed concerns about the accuracy of information, and nearly one-third reported modifying medication-related decisions on the basis of ChatGPT’s advice, highlighting potential patient safety risks (24). Similarly, Al Suwaidan et al. demonstrated that individuals with prior experience using ChatGPT reported more favorable perceptions of its usefulness for health-related purposes than nonusers did. Moreover, concerns about misinformation, privacy, and reliability persist (25). Together, these studies suggest the growing public acceptance of generative AI in healthcare but also underscore the importance of ensuring that users understand its limitations and continue to rely on healthcare professionals for clinical decision-making.

Despite the increasing number of studies investigating public use and perceptions of ChatGPT in healthcare, important gaps remain in the integrated assessment of these domains. Previous Saudi studies have focused primarily on medication-related use (24) or compared perceptions between users and nonusers (25). However, the available evidence has not comprehensively examined knowledge, attitudes, perceptions, risk–reward appraisal, and sociodemographic characteristics together within a single survey assessing reported health-related ChatGPT use. By integrating established technology acceptance frameworks with behavioral and sociodemographic factors, this study provides a broader understanding of the factors influencing the public adoption of generative AI for healthcare. These findings may contribute to the development of evidence-based digital health literacy initiatives, inform future regulatory policies, and support the responsible integration of generative artificial intelligence into healthcare systems. Therefore, this study aimed to assess reported use of ChatGPT for health-related purposes and examine its associations with demographic characteristics, self-rated health literacy, knowledge, attitudes, perceptions, and risk–reward appraisal among adults recruited from the Western Region of Saudi Arabia.

## 2 Methods

### 2.1 Study design and setting

This study utilized a cross-sectional, questionnaire-based design to assess the use of ChatGPT for health-related purposes and its associated factors. Data were collected from July 4 to November 20, 2025, using an online survey distributed to the general population in the Western Region of Saudi Arabia. This cross-sectional study was designed and reported in accordance with the Strengthening the Reporting of Observational Studies in Epidemiology (STROBE) Statement to ensure transparent and comprehensive reporting of observational research (Supplementary Table 1).

### 2.2 Study population and sampling

Eligible participants were adults aged 18 years and above who were able to read and understand the survey language and consent to participate. Participants with incomplete questionnaires were excluded from the final analysis before the statistical analysis. A nonprobability convenience sampling technique was used to recruit participants through online platforms, including social media channels and electronic communication networks.

### 2.3 Sample size calculation

The minimum sample size was calculated using a single-population proportion formula, assuming a 50% expected prevalence of ChatGPT use for health-related purposes, a 95% confidence level, and a 5% margin of error. The calculation yielded a minimum required sample of 384 participants. No specific nonresponse percentage was prespecified in the sample-size calculation. The final analytical sample consisted of 735 participants who completed the questionnaire and met the eligibility criteria.

The minimum sample size was calculated using the single-population proportion formula:

$$n=Z^{2}p(1-p)/d^{2}$$

where n is the required sample size, Z is the standard normal deviate corresponding to a 95% confidence level (1.96), p is the expected prevalence (0.50), and d is the desired margin of error (0.05). Thus:

$$n={\left(1.96\right)}^{2}(0.50)(1–0.50)/{\left(0.05\right)}^{2}\approx 384$$

The final analytical sample comprised 735 participants with complete questionnaires.

### 2.4 Data collection instrument

Data were collected via a structured, self-administered questionnaire. The questionnaire was developed following an extensive review of the literature on public acceptance of artificial intelligence and digital health technologies. Individual items were informed by previously described constructs from the technology acceptance model (TAM) (19) and selected domains of the unified theory of acceptance and use of technology (UTAUT) (20). These frameworks were used to guide questionnaire development rather than to formally test their theoretical models. The final questionnaire was reviewed by experts for content validity and is provided in Supplementary material S1 to facilitate transparency and reproducibility. The questionnaire is available in both English and Arabic to accommodate the linguistic diversity of the target population. The questionnaire consisted of five main sections:

1. The demographic characteristics included age, sex, nationality, marital status, education level, employment status, presence of chronic conditions, smoking status, and self-rated health literacy.
2. ChatGPT use: participants were asked whether they had ever used ChatGPT for health-related purposes (yes/no).
3. Knowledge: Four items assessing awareness of ChatGPT limitations in healthcare, informed by the literature on patients’ understanding of online health information.
4. Attitudes: Five items evaluating participants’ perspectives toward using ChatGPT for health information. Adapted from the technology acceptance model (TAM), which evaluates perceived usefulness and ease of use (19).
5. Perceptions: Seven items assessing perceived usefulness and effectiveness. The constructs of perceived usefulness and performance expectancy are based on the unified theory of acceptance and use of technology (UTAUT) (20).
6. Risk–reward appraisal: 4 items evaluating perceived risks and benefits.

All items in the knowledge, attitudes, perceptions, and risk–reward domains were measured via a 5-point Likert scale ranging from 1 (strongly disagree) to 5 (strongly agree). Negatively worded items were reverse-coded before analysis.

### 2.5 Instrument validity and reliability

Content validity was established through two expert reviews by professionals in healthcare and research methodology. A pilot study was conducted on a small sample (*N* = 20) to assess clarity and comprehensibility; however, these data were not included in the analysis. The internal consistency reliability of the scales was assessed using Cronbach’s alpha. The reliability values were acceptable to high: knowledge (α = 0.81), attitudes (α = 0.77), perceptions (α = 0.92), and risk–reward appraisal (α = 0.63). The risk–reward appraisal scale demonstrated comparatively lower internal consistency (Cronbach’s α = 0.63). Because the items addressed both perceived benefits and potential risks, this coefficient should be interpreted cautiously and does not establish strong unidimensionality. These findings provide information on internal consistency but do not constitute comprehensive psychometric validation of the instrument.

### 2.6 Data collection procedure

The survey was distributed electronically via online platforms (e.g., X, WhatsApp, and LinkedIn), university email lists, and community centers in major cities in the Western Region (Taif, Jeddah, Makkah, and Jazan). These locations represented recruitment sites rather than probability-based sampling strata, and no population-based sampling quotas were applied. Participation was voluntary, and informed consent was obtained from all participants before they completed the questionnaire. Responses were collected anonymously to ensure confidentiality. Because recruitment was conducted through multiple online and community-based channels, a single denominator for the number of individuals exposed to the survey invitation was not available. The final analytical sample consisted of 735 participants who completed the questionnaire and met the eligibility criteria. Incomplete questionnaires were excluded before analysis.

### 2.7 Variables and measures

The primary outcome variable was the use of ChatGPT for health-related purposes (yes/no). Specific health-related applications of ChatGPT were assessed descriptively and were not included as separate outcomes in the multivariable regression model. Demographic characteristics, self-rated health literacy, and composite scores for knowledge, attitudes, perceptions, and risk–reward appraisal were the independent variables. Scale scores were computed by summing item responses within each domain. Higher scores reflect higher levels of knowledge, positive attitudes, perceptions, or perceived benefits relative to risk. Self-rated health literacy was assessed using a single self-reported question in which participants categorized their perceived health literacy as low, moderate, or high. This item was intended to capture perceived rather than objectively measured health literacy.

### 2.8 Statistical analysis

Descriptive statistics (frequencies, percentages, means, and standard deviations) were calculated to summarize participant characteristics and item responses. The internal consistency reliability of multi-item scales (knowledge, attitudes, perceptions, and risk–reward appraisal) was assessed via Cronbach’s α. Scale scores were computed by summing item responses, and group differences in mean scores were examined using independent-samples t tests and one-way ANOVA, as appropriate. Associations between categorical variables and ChatGPT use for health-related purposes were initially explored using chi-square tests of independence. To further evaluate the factors associated with ChatGPT use for health-related purposes, binary logistic regression analyses were conducted. Both unadjusted and adjusted models were estimated, with odds ratios (ORs) and 95% confidence intervals (CIs) reported. The adjusted model included the demographic characteristics, self-rated health literacy, and composite knowledge, attitude, perception, and risk–reward appraisal scores specified *a priori* for the analysis. The results are presented as associations rather than causal or predictive effects. All analyses were performed via IBM SPSS Statistics (version 24). Statistical significance was set at *p* < 0.05. Only complete questionnaires were included in the final analysis; therefore, no imputation methods for missing data were needed.

**Table 4:** Binary logistic regression for predicting ChatGPT use for health-related purposes: unadjusted and adjusted models.

Variable	*Unadjusted* OR [95% CI]	*p*	*Adjusted* OR [95% CI]	*p*
Age group				
18–24	6.71 (4.45–10.13)	<0.001	5.37 (2.34–12.34)	<0.001
25–44	2.71 (1.82–4.02)	<0.001	2.28 (1.32–3.96)	0.003
≥45	Ref		Ref	
Sex				
Female	0.90 (0.65–1.24)	0.507	1.13 (0.74–1.74)	0.566
Male	Ref		Ref	
Nationality				
Saudi	1.68 (0.98–2.87)	0.056	1.35 (0.71–2.55)	0.358
Non-Saudi	Ref		Ref	
Marital status				
Single	3.60 (1.75–7.41)	0.001	0.77 (0.28–2.12)	0.619
Married	1.25 (0.61–2.57)	0.542	1.04 (0.43–2.51)	0.925
Divorced/Widowed	Ref		Ref	
Educational level				
≤ High school\diploma	Ref	0.023	Ref	0.011
Bachelor’s degree	1.46 (1.05–2.03)	0.008	1.82 (1.15–2.89)	0.006
Higher education	2.05 (1.20–3.49)		2.80 (1.34–5.85)	
Employment				
Student	5.26 (3.44–8.05)	<0.001	2.56 (1.29–5.09)	0.007
Employed	1.60 (1.11–2.31)	0.013	1.19 (0.71–1.99)	0.515
Unemployed/Retired	Ref		Ref	
Having chronic conditions				
Yes	0.75 (0.49–1.14)	0.173	1.75 (0.97–3.14)	0.063
No	Ref		Ref	
Self-rated health literacy				
Low	Ref	0.072	Ref	0.416
Moderate	1.71 (0.95–3.07)	<0.001	1.35 (0.66–2.78)	0.101
High	3.19 (1.73–5.90)		1.91 (0.88–4.14)	
Total knowledge score	0.97 (0.92–1.03)	0.334	1.02 (0.95–1.10)	0.522
Total attitude score	1.27 (1.21–1.33)	<0.001	1.05 (0.96–1.14)	0.330
Total perceptions score	1.22 (1.18–1.26)	<0.001	1.19 (1.12–1.26)	<0.001
Total risk–reward appraisal score	1.33 (1.25–1.41)	<0.001	0.98 (0.89–1.09)	0.751

### 2.9 Ethical considerations

The study was conducted in accordance with the ethical principles for research involving human subjects. This study was approved by Taif University (approval number: 46–350, date: 06--24-2025). Informed consent was obtained electronically, and participation was voluntary. The participants were required to provide electronic informed consent before accessing the questionnaire. Data were collected anonymously and were used for research purposes only.

## 3 Results

### 3.1 Participant characteristics

A total of 735 participants were included in the analysis. Of these, 487 (66.3%) reported having used ChatGPT for health-related purposes, whereas 248 (33.7%) had not. As shown in Table 1, ChatGPT use varied significantly across several demographic factors. Younger participants were more likely to use ChatGPT, with 82.3% of those aged 18–24 reporting use, compared with 41.0% among those aged ≥45 (*p* < 0.001). Marital status was also associated with use, with single participants demonstrating the highest usage rate (77.2%) compared with married (54.1%) and divorced/widowed individuals (48.5%; *p* < 0.001; Table 1).

**Table 1:** Demographic characteristics of the participants and their associations with ChatGPT use for health-related purposes (*N* = 735).

Variable	Total n (%)	Ever used ChatGPT for health-related purposes?		*p*
		No (*n* = 248)	Yes (*n* = 487)	
Age group				<0.001
18–24	311 (42.3%)	55 (17.7%)	256 (82.3%)	
25–44	236 (32.1%)	82 (34.7%)	154 (65.3%)	
≥45	188 (25.6%)	111 (59.0%)	77 (41.0%)	
Sex				0.507
Female	468 (63.7%)	162 (34.6%)	306 (65.4%)	
Male	267 (36.3%)	86 (32.2%)	181 (67.8%)	
Nationality				0.054
Saudi	675 (91.8%)	221 (32.7%)	454 (67.3%)	
Non-Saudi	60 (8.2%)	27 (45.0%)	33 (55.0%)	
Marital status				<0.001
Single	395 (53.7%)	90 (22.8%)	305 (77.2%)	
Married	307 (41.8%)	141 (45.9%)	166 (54.1%)	
Divorced/Widowed	33 (4.5%)	17 (51.5%)	16 (48.5%)	
Educational level				0.010
≤ High school\diploma	261 (35.5%)	105 (40.2%)	156 (59.8%)	
Bachelor’s degree	381 (51.8%)	120 (31.5%)	261 (68.5%)	
Higher education	93 (12.7%)	23 (24.7%)	70 (75.3%)	
Employment				<0.001
Student	267 (36.3%)	43 (16.1%)	224 (83.9%)	
Employed	261 (35.5%)	101 (38.7%)	160 (61.3%)	
Unemployed/Retired	207 (28.2%)	104 (50.2%)	103 (49.8%)	
Having chronic conditions				0.172
Yes	109 (14.8%)	43 (39.4%)	66 (60.6%)	
No	626 (85.2%)	205 (32.7%)	421 (67.3%)	
Smoking				0.688
Yes	94 (12.8%)	30 (31.9%)	64 (68.1%)	
No	641 (87.2%)	218 (34.0%)	423 (66.0%)	
Self-rated health literacy				<0.001
Low	51 (6.9%)	26 (51.0%)	25 (49.0%)	
Moderate	407 (55.4%)	154 (37.8%)	253 (62.2%)	
High	277 (37.7%)	68 (24.5%)	209 (75.5%)	

Values are presented as n (%). Associations between categorical variables and ChatGPT use were assessed using the chi-square test of independence. A two-sided *p*-value < 0.05 was considered statistically significant. *p* < 0.05 indicates statistical significance.

Educational level showed a significant association, as participants with higher education reported greater use (75.3%) than did those with a high school education or less (59.8%) (*p* = 0.010). Employment status was another significant factor; students had the highest usage rate (83.9%), followed by employed individuals (61.3%) and unemployed/retired participants (49.8%) (*p* < 0.001). Self-rated health literacy was strongly associated with ChatGPT use (*p* < 0.001), with individuals reporting high health literacy demonstrating the greatest usage (75.5%). No significant associations were found for sex (*p* = 0.507), nationality (*p* = 0.054), chronic conditions (*p* = 0.172), or smoking (*p* = 0.688; Table 1).

### 3.2 Knowledge, attitudes, perceptions, and risk–reward appraisal toward ChatGPT use

Overall, the participants demonstrated a high level of knowledge about the limitations of ChatGPT in medical contexts. The majority agreed that ChatGPT is not specifically designed for medical use and that its health-related advice may not always be accurate. Nearly all the participants also acknowledged the importance of consulting healthcare professionals for accurate diagnosis and safe medication use (mean = 4.58 ± 0.74; Table 2).

**Table 2:** Distribution of responses to the knowledge, attitudes, perceptions, and risk–reward items regarding ChatGPT use for health-related purposes.

Items	(1) Strongly Disagree %	(2) Disagree %	(3) Neutral %	(4) Agree %	(5) Strongly Agree %	Mean ± SD
Knowledge						
I understand that ChatGPT is not specifically designed for medical or health-related purposes.	1.4	4.8	17.0	35.5	41.4	4.11 ± 0.94
I am aware that ChatGPT’s health-related advice may not always be accurate or verified by medical professionals.	1.1	3.4	13.5	36.9	45.2	4.22 ± 0.88
I know that using ChatGPT for self-diagnosis or medication advice could lead to incorrect health decisions.	1.1	3.8	14.4	37.0	43.7	4.18 ± 0.89
I know that consulting a healthcare professional is necessary for an accurate diagnosis and safe medication use	0.8	1.4	6.1	22.4	69.3	4.58 ± 0.74
Attitudes						
ChatGPT can provide helpful health information to support my decisions.	3.9	9.1	21.6	42.6	22.7	3.71 ± 1.04
I feel confident using ChatGPT to learn about health conditions or medications without consulting a healthcare professional.	12.1	33.3	27.5	18.2	8.8	2.78 ± 1.14
I think using ChatGPT for self-diagnosis is convenient because it is accessible anytime and anywhere.	8.3	17.0	22.4	34.6	17.7	3.36 ± 1.19
I am worried that using ChatGPT for self-diagnosis or medication advice could lead to harmful health outcomes. *	27.1	32.9	22.3	15.4	2.3	2.33 ± 1.10
I believe ChatGPT is a trustworthy source for health information compared to other online sources	8.6	17.8	30.5	28.4	14.7	3.23 ± 1.16
Perceptions						
ChatGPT can help me achieve my goals when seeking health information or medication advice.	4.5	9.4	24.9	44.2	17.0	3.60 ± 1.02
Using ChatGPT reduces the effort needed to find information about health conditions or medications.	3.1	6.5	18.8	47.3	24.2	3.83 ± 0.97
I have successfully obtained useful health or medication information from ChatGPT in the past.	9.0	12.2	18.0	39.0	21.8	3.52 ± 1.21
I am satisfied with ChatGPT’s ability to provide health-related information or medication advice.	7.5	17.0	29.1	32.0	14.4	3.29 ± 1.13
ChatGPT helps me make informed and timely decisions about my health or medication use.	16.7	31.7	25.2	18.1	8.3	2.70 ± 1.19
I am willing to make health-related decisions based on ChatGPT’s recommendations.	8.3	13.6	31.4	32.5	14.1	3.31 ± 1.13
ChatGPT provides clear and understandable recommendations for health and medication decisions.	9.5	20.4	29.5	28.6	12.0	3.13 ± 1.16
Risk–Reward Appraisal						
The benefits of using ChatGPT for self-diagnosis or medication advice outweigh the potential risks.	10.1	25.9	31.4	23.3	9.4	2.96 ± 1.13
I am worried that using ChatGPT for self-diagnosis or medication advice could lead to harmful health outcomes. *	2.4	10.7	23.9	39.2	23.7	2.29 ± 1.02
I trust ChatGPT to provide safe and reliable advice for nonprescription medications (OTC).	9.9	23.4	26.4	28.8	11.4	3.08 ± 1.17
I believe the risks of using ChatGPT for health advice are lower than consulting unverified online sources.	3.8	9.5	26.5	42.2	18.0	3.61 ± 1.01

*Negatively worded items are presented in their original response direction in this table. Thus, higher agreement with these items indicates greater concern about potential harm. Item-level percentages and means should therefore be interpreted according to the wording of each item”.

The attitudinal responses were more variable. While many participants agreed that ChatGPT can provide helpful health information (mean = 3.71 ± 1.04), fewer expressed confidence in using ChatGPT independently for health information or self-diagnosis without consulting a healthcare professional (mean = 2.78 ± 1.14). Additionally, participants expressed moderate levels of concern regarding potential harmful outcomes associated with using ChatGPT for self-diagnosis or medication advice (Table 2).

The participants generally perceived ChatGPT as a useful tool for achieving health information-seeking goals (mean = 3.60 ± 1.02) and for reducing the effort required to locate health information (mean = 3.83 ± 0.97). However, participants reported limited willingness to base health-related decisions directly on ChatGPT recommendations (mean = 2.70 ± 1.19; Table 2).

The risk–reward appraisal scores suggested a cautious attitude toward the use of ChatGPT in health contexts. The participants were moderately likely to believe that the benefits of using ChatGPT for health advice outweighed potential risks (mean = 2.96 ± 1.13) while also expressing concerns about possible adverse health outcomes (Table 3).

### 3.3 Scale reliability and descriptive statistics

The internal consistency estimates varied across the study scales. The Cronbach’s α coefficients were 0.81 for the knowledge scale, 0.77 for attitudes, and 0.92 for perceptions, indicating good internal consistency. The risk–reward appraisal scale showed lower consistency compared with the other scales (α = 0.63). The mean scale scores were as follows: knowledge (17.1 ± 2.8), attitudes (15.4 ± 4.1), perceptions (23.4 ± 6.4), and risk–reward appraisal (11.9 ± 2.9; Table 3).

**Table 3:** Descriptive statistics and reliability of study scales assessing ChatGPT use for health-related purposes.

Scale	Possible range	Mean ± SD	Cronbach’s α
Knowledge	4–20	17.1 ± 2.8	0.81
Attitudes	5–25	15.4 ± 4.1	0.77
Perceptions	7–35	23.4 ± 6.4	0.92
Risk–Reward Appraisal	4–20	11.9 ± 2.9	0.63

Composite scale scores were calculated by summing the item responses within each domain. Higher scores indicate higher levels of knowledge, more positive attitudes, more favorable perceptions, or greater perceived benefits relative to risks, as applicable. Mean values are presented as mean ± standard deviation. Cronbach’s α represents the internal consistency reliability of each multi-item scale. The risk–reward appraisal scale demonstrated comparatively lower internal consistency (α = 0.63) and should therefore be interpreted cautiously.

### 3.4 Comparison of mean knowledge, attitudes, perceptions, and risk–reward scores by ChatGPT use for health-related purposes

Compared with nonusers, participants who used ChatGPT for health-related purposes had significantly higher mean scores for attitudes, perceptions, and risk–reward appraisals (all *p* < 0.01). The greatest difference was observed in the perceptions domain. In contrast, knowledge scores did not differ significantly between the two groups. These findings indicate that the use of ChatGPT is associated with more favorable attitudes and perceived benefits but not with differences in knowledge (Figure 1).

**Figure 1:**
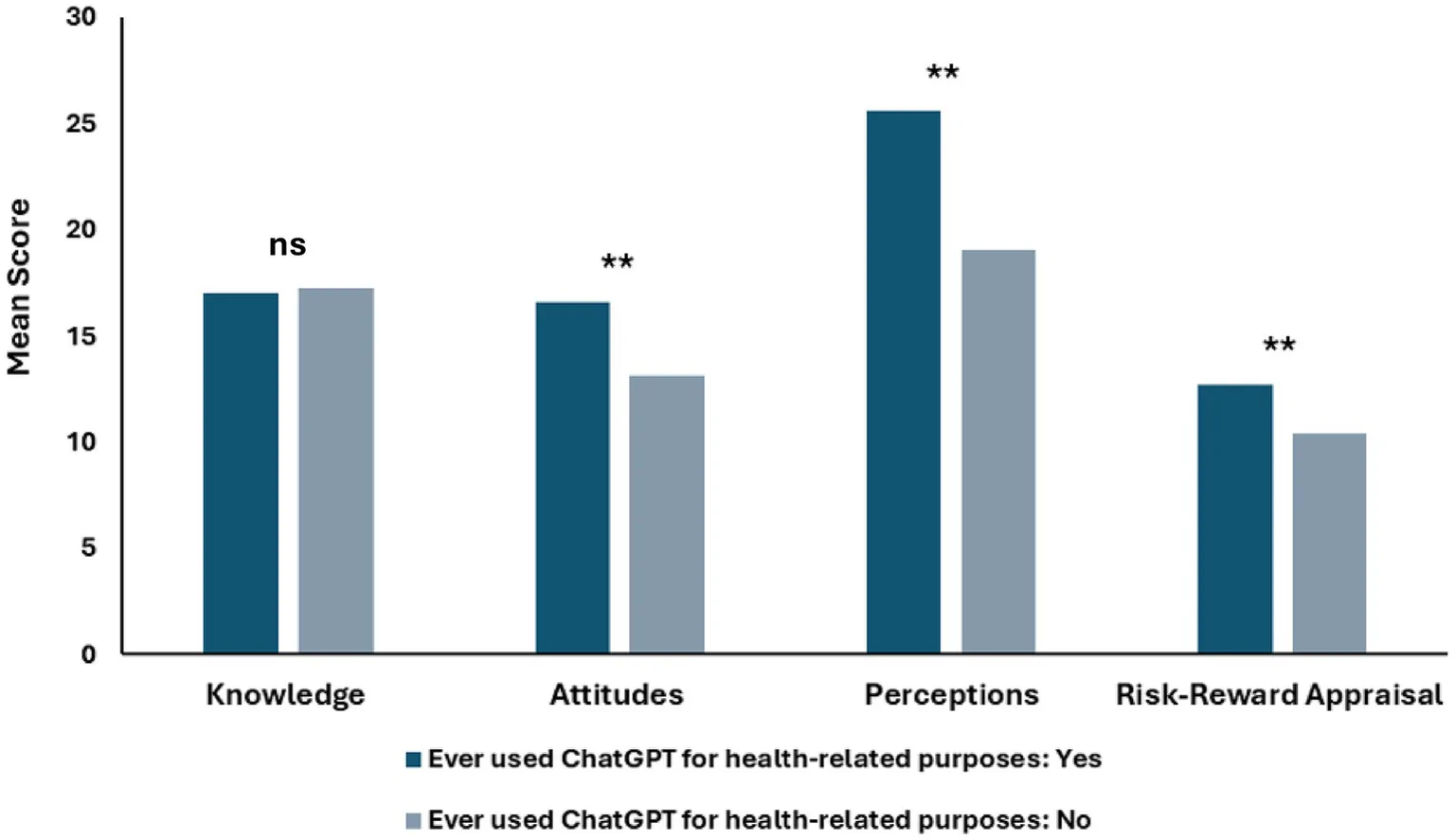
Comparison of mean knowledge, attitude, perception, and risk–reward appraisal scores between participants who reported using and not using ChatGPT for health-related purposes. Values are presented as mean ± SD. Independent-samples t-tests were used for comparisons between the two groups. **p < 0.01; ns, not statistically significant (*p* ≥ 0.05).

### 3.5 Factors associated with ChatGPT use for health-related purposes

Binary logistic regression analyses were performed to examine factors associated with ChatGPT use for health-related purposes (Table 4). In the adjusted (multivariable) model, controlling for all variables, younger age, higher education, and student status remained independent predictors of use. Compared with those aged ≥45 years, those aged 18–24 years were 5.37 times more likely (aOR = 5.37, 95% CI = 2.34–12.34, *p* < 0.001), and those aged 25–44 years were 2.28 times more likely (aOR = 2.28, 95% CI = 1.32–3.96, *p* = 0.003) to have used ChatGPT for health purposes. Bachelor’s degree holders (aOR = 1.82, 95% CI = 1.15–2.89, *p* = 0.011) and postgraduate-educated individuals (aOR = 2.80, 95% CI = 1.34–5.85, *p* = 0.006) presented significantly greater odds than did those with high school/diploma education. Compared with unemployed/retired individuals, students were 2.56 times more likely to use ChatGPT for health purposes (aOR = 2.56, 95% CI = 1.29–5.09, *p* = 0.007). The total perception score remained significantly associated with the outcome (aOR = 1.19, 95% CI: 1.12–1.26, *p* < 0.001), indicating an independent effect after adjustment for potential confounders. Other variables, including sex, nationality, marital status (after adjustment), chronic conditions, health literacy, knowledge, attitudes, and a higher risk–reward appraisal score, were not independently significant. These findings indicate substantial adoption of ChatGPT for health-related inquiries among younger, more educated, and student populations in this sample, which was determined particularly by favorable perceptions, despite widespread recognition of the tool’s limitations and the importance of professional medical consultation.

### 3.6 Intention to use ChatGPT for health-related applications

Supplementary Table 2 and Figure 2 present the participants’ intentions to use ChatGPT for health-related purposes. The highest level of acceptance was observed for using ChatGPT to seek information about medications, with 59.1% of respondents reporting that they were likely or very likely to use it for this purpose (45.0 and 14.1%, respectively), yielding the highest mean score among all items (3.44 ± 1.13). With respect to the use of ChatGPT as a primary source of health-related information in the future, 37.0% of the participants indicated that they were likely or very likely to do so, whereas an equal proportion (36.9%) reported being unlikely or very unlikely. The mean score for this item was 3.00 ± 1.22, reflecting a neutral overall attitude. When asked about self-diagnosing health conditions, participants were generally hesitant: 45.7% reported they were unlikely or very unlikely to use ChatGPT for this purpose, whereas 29.9% were likely or very likely to do so. This item had a mean score of 2.78 ± 1.20.

**Figure 2:**
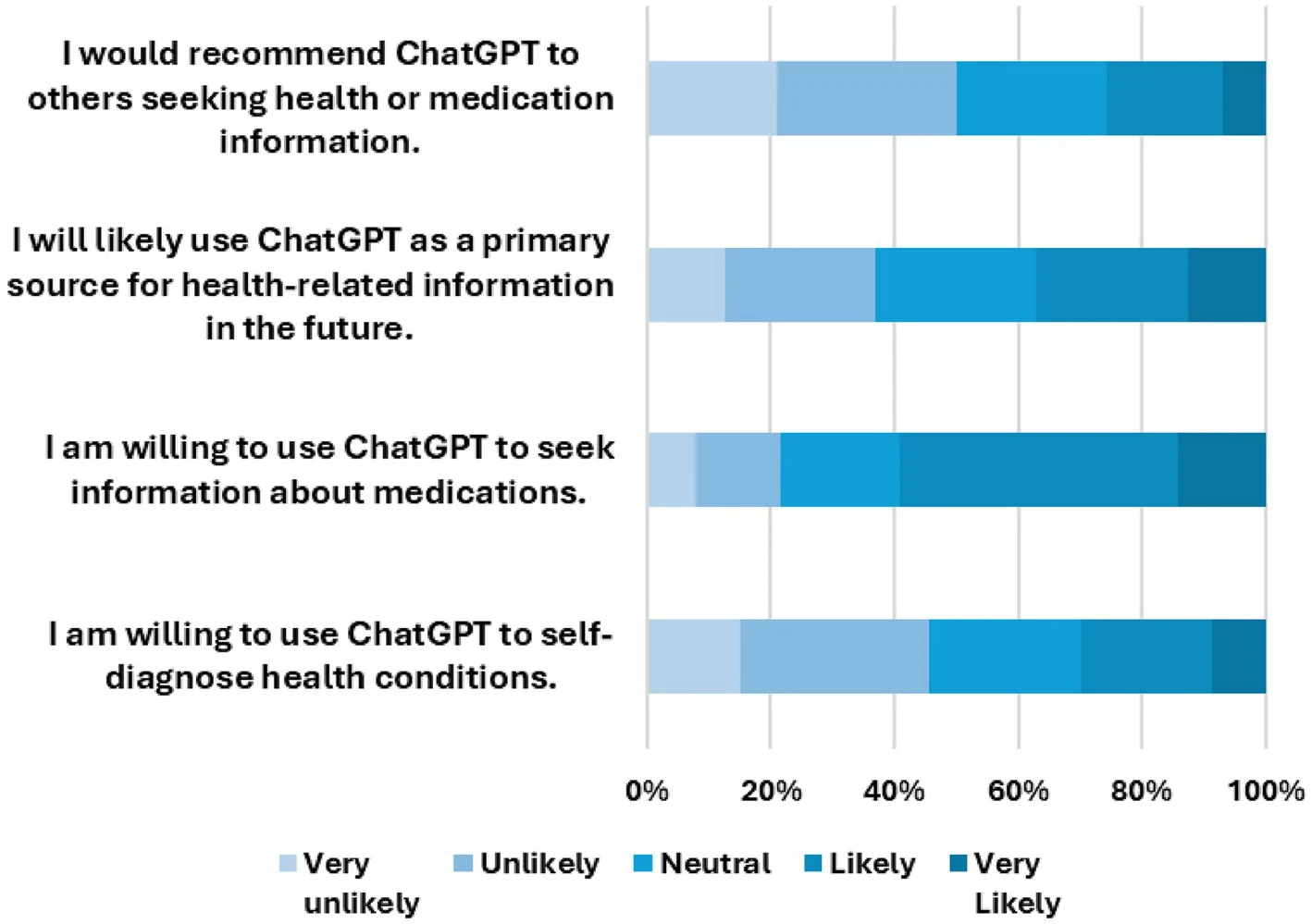
Intention to use ChatGPT for health-related purposes.

Similarly, the respondents showed limited willingness to recommend ChatGPT to others seeking health or medication information. Half of the participants (50.0%) were unlikely or very unlikely to recommend it, whereas only 25.7% were likely or very likely to do so. This item had the lowest mean score (2.62 ± 1.20; Supplementary Table 2; Figure 2). Overall, the participants demonstrated the greatest willingness to use ChatGPT to obtain medication-related information while showing more cautious attitudes toward self-diagnosis and toward relying on ChatGPT as a primary health information source and recommending it to others.

### 3.7 Specific health-related uses of ChatGPT

Supplementary Table 3 and Figure 3 summarize the frequency of specific health-related uses of ChatGPT among participants. Overall, ChatGPT was most frequently used to seek advice on beauty products and vitamins or dietary supplements, whereas contraceptives, smoking cessation medications, and allergy medications were among the least frequently consulted topics.

**Figure 3:**
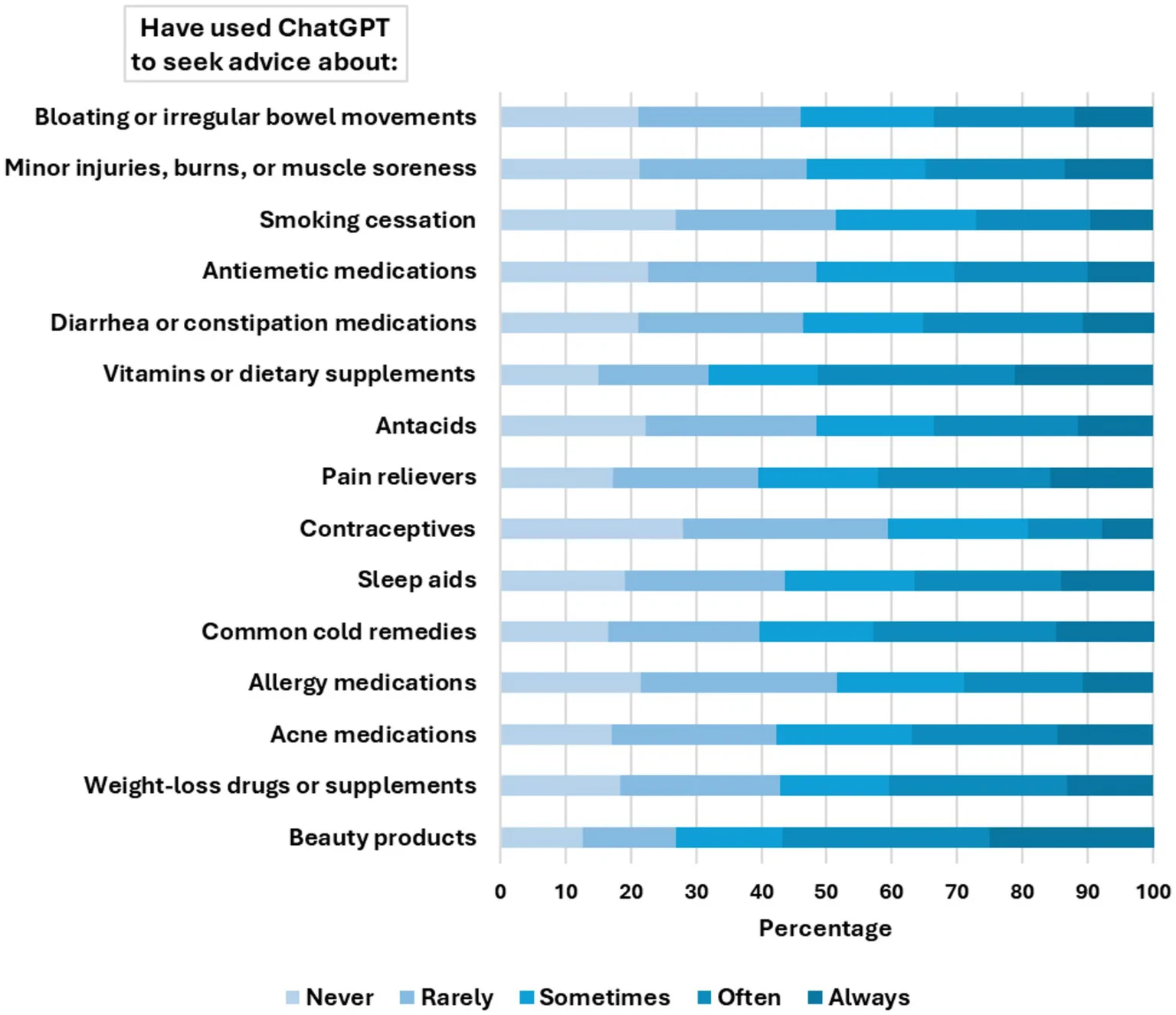
Frequency of specific uses of ChatGPT for health-related purposes.

The most commonly reported use was seeking advice about beauty products, with 56.9% of participants reporting that they often or always used ChatGPT for this purpose (31.7% often and 25.2% always). Similarly, more than half of the participants reported often or always using ChatGPT to obtain information about vitamins or dietary supplements (51.4%), making it the second most common application. Moderate levels of use were observed for common cold remedies (42.9%), pain relievers (42.2%), weight-loss drugs or supplements (40.5%), acne medications (36.9%), sleep aids (36.6%), and medications for diarrhea or constipation (35.4%), where participants reported often or always seeking advice through ChatGPT (Supplementary Table 3; Figure 3).

Lower frequencies of use were reported for allergy medications (28.9%), antacids (33.5%), antiemetic medications (30.6%), medications used to reduce bloating or irregular bowel movements (33.6%), and medications used to prevent localized pain from minor injuries, burns, or muscle soreness (34.8%). In addition, advice regarding smoking cessation medications was relatively uncommon, with only 27.0% reporting often or always using ChatGPT for this purpose, whereas 51.4% reported never or rarely doing so. The least frequently reported use was seeking advice about contraceptives. Nearly two-thirds of the respondents (59.3%) indicated that they never or rarely used ChatGPT for contraceptive-related information, whereas only 19.2% reported using it often or always (Supplementary Table 3; Figure 3).

## 4 Discussion

The current study provides one of the most comprehensive evaluations of public use of ChatGPT for health purposes in the western region of Saudi Arabia, investigating knowledge, attitudes, perceptions, risk–reward appraisals, and factors related to adoption simultaneously. Approximately two-thirds of the recruited participants reported that they had used ChatGPT to access health-related information, suggesting that generative artificial intelligence has quickly emerged as an important health information source for the general population. While the participants showed good knowledge of the limitations of ChatGPT and the ongoing essential role of healthcare professionals in diagnostic and treatment decisions, many continued to view ChatGPT as a helpful and convenient resource. The results indicate an increasing reliance on conversational AI, which is more motivated by perceived usefulness and accessibility than by absolute confidence in its medical reliability. These results are consistent with those reported by Nadarzynski et al., who reported that although most individuals are receptive to AI-powered health chatbots, concerns about the technology may limit their adoption. These findings emphasize the importance of a user-centered design that addresses users’ concerns and improves trust and usability (26).

Our finding that a high proportion of participants reported using ChatGPT is consistent with the growing international literature on the rapid uptake of generative AI by the public for health-related purposes. Users have been increasingly using ChatGPT to learn about symptoms, medications, lab results, and preventive health before consulting healthcare professionals, as shown in previous studies (27, 28). In addition, our results are consistent with previous Saudi studies that have demonstrated increasing acceptance of ChatGPT for health-related purposes, while highlighting persistent concerns about the accuracy, reliability, and privacy of AI-generated information (24, 25). Overall, these findings suggest that, despite ongoing uncertainty about the accuracy of AI-generated medical advice, public acceptance of generative AI is increasing.

A key finding of our study is that the participants demonstrated a good understanding of the limitations of ChatGPT. Most respondents recognized that ChatGPT is not intended to replace clinical care and agreed that healthcare professionals remain essential for making diagnostic and treatment decisions. This finding is consistent with previous research showing that users continue to rely on ChatGPT for its speed, accessibility, and ease of communication while acknowledging that AI-generated responses may occasionally be inaccurate (15, 29). Nov et al. reported that users are generally willing to trust ChatGPT for low-risk health-related questions. However, concerns about the accuracy and reliability of its responses continue to affect their trust (30). Evidence from evaluations of the clinical performance of ChatGPT further supports these concerns. For example, ChatGPT has been shown to provide satisfactory answers to common patient questions about hip arthroscopy; however, inaccuracies were identified in several responses, leading the authors to recommend caution when using ChatGPT for patient education (31). Collectively, these findings suggest that awareness of the limitations of ChatGPT does not necessarily discourage its use. Instead, users appear to balance its recognized shortcomings against its convenience, using it as a readily accessible source of health information while continuing to value professional medical advice for clinical decision-making.

Although knowledge scores were comparable between users and nonusers, participants who had previously used ChatGPT demonstrated significantly more positive attitudes, perceptions, and risk–reward appraisals. These findings indicate that previous ChatGPT use was associated with more favorable attitudes, perceptions, and risk–reward appraisal scores, whereas knowledge scores did not differ significantly between users and nonusers. Similar results have been reported in previous studies where perceived usefulness, perceived ease of use and trust were found to be stronger determinants of technology adoption than objective knowledge (16, 32–34). This pattern also aligns with the unified theory of acceptance and use of technology (UTAUT) and the technology acceptance model (TAM), which state that behavioral intention is primarily determined by perceived usefulness and performance expectancy (19, 20). Our findings are consistent with the key concepts of the TAM and UTAUT. Specifically, perceived usefulness and positive perceptions appeared to play important roles in ChatGPT adoption. However, because this study did not evaluate all the theoretical constructs or use structural equation modeling, these frameworks should be considered conceptual guides rather than formally tested models.

In our multivariable analysis, younger age, higher educational attainment, and being a student were independent factors associated with ChatGPT use for health-related purposes. This aligns with previous research reporting that younger adults and university students are early adopters of emerging digital technologies because they have greater digital literacy, familiarity with artificial intelligence, and confidence in online information seeking (15, 25, 35). Higher education beyond a high school diploma may also help people formulate more effective prompts and critically analyze the responses generated by AI, thereby promoting the adoption of AI.

The pattern of health-related ChatGPT use observed in our study provides additional insight into how the public currently engages with generative AI. Participants most frequently sought advice on beauty products, vitamins, and dietary supplements, whereas consultation on birth control or smoking cessation medications was considerably less common. This pattern suggests that users are more inclined to rely on ChatGPT for topics perceived as lower risk, while remaining more cautious about issues that require individualized clinical assessment. Similar findings have been reported previously, with users demonstrating greater trust in ChatGPT for general health and wellness questions than for diagnosis or treatment decisions (30). Nevertheless, the frequent use of ChatGPT for medication-related information remains clinically important because inaccurate recommendations regarding drug selection, dosing, contraindications, or interactions may contribute to inappropriate self-medication or delayed consultation with healthcare professionals (36). The clinical implications of these uses should be interpreted according to the type of health-related information sought. Requests concerning beauty products and general wellness may generally involve lower immediate clinical risk, whereas questions about medications, weight-loss products, dietary supplements, and sleep aids may carry greater risks when AI-generated information is used without professional verification. In such contexts, inaccurate or incomplete information could contribute to inappropriate dosing, contraindications, drug–drug or drug–supplement interactions, inappropriate self-medication, or delayed consultation with healthcare professionals. Therefore, the reported frequency of ChatGPT use should not be interpreted as evidence that participants acted on its recommendations or experienced medication-related outcomes. Rather, these findings highlight the importance of distinguishing general health-information seeking from health-related decisions that require individualized clinical assessment.

Overall, our results indicate that the public adoption of ChatGPT is a balance between perceived benefits and recognized limitations. Healthcare systems should not seek to discourage the use of AI; rather, they should focus on improving digital health literacy and educating the public about when ChatGPT can be a useful educational tool and when professional medical evaluation is needed. Future longitudinal studies should examine how generative AI, as it continues to advance, affects clinical decision-making, patient safety, healthcare utilization, medication adherence, and patient–physician communication.

### 4.1 Strengths and limitations

This study has several notable strengths. First, it included a relatively large sample size (*N* = 735), exceeding the minimum required sample size, thereby enhancing the statistical power and precision of the findings. Second, the study assessed multiple dimensions of ChatGPT use, including knowledge, attitudes, perceptions, and risk–reward appraisals, providing a more nuanced understanding of user behavior. Third, the questionnaire was developed on the basis of established technology acceptance frameworks, including the TAM (19), and selected constructs from the UTAUT (20, 37), which enhanced the conceptual foundation of the instrument. Furthermore, the questionnaire demonstrated good to excellent internal consistency across most domains, supporting the reliability of the measured constructs. Finally, the use of multivariable logistic regression allowed the identification of independent factors associated with ChatGPT use for health-related purposes while controlling for potential confounders.

Despite these strengths, several limitations should be considered. First, the cross-sectional design limits the ability to establish causal relationships between variables. Additionally, the use of a convenience sampling method may introduce selection bias and limit the generalizability of the findings to the broader population. Because recruitment used a nonprobability convenience sampling approach, the sample should not be considered representative of adults in the Western Region of Saudi Arabia. The observed proportion of ChatGPT use therefore describes the recruited sample rather than a population prevalence estimate. Although recruitment was conducted across several cities, the study did not use city-level probability sampling or predefined population-based quotas. Therefore, the relative contribution of individual cities to the final sample and the representativeness of the sample across the broader Western Region cannot be assumed. Participants were recruited using convenience sampling through online platforms; individuals who are younger, more digitally literate, and more familiar with emerging technologies may have been overrepresented. Consequently, the reported prevalence of ChatGPT use may be higher than that in the general population. Therefore, caution should be employed when these findings are generalized beyond the study population.

A detailed response rate could not be calculated because recruitment was conducted through multiple electronic and community-based channels and the number of individuals exposed to the survey invitation was not systematically recorded. Consequently, the potential magnitude of nonresponse and self-selection bias cannot be quantified. The data were self-reported, which may be subject to recall bias and social desirability bias, particularly with respect to health behaviors and perceptions. Health literacy was assessed using a single self-rated item rather than a validated multidimensional health literacy instrument. Consequently, the measure reflects participants’ perceived health literacy and may be influenced by self-perception, social desirability, and misclassification. Internal consistency reliability cannot be assessed for a single-item measure. Although the questionnaire underwent expert review, pilot testing for clarity, and internal consistency assessment, comprehensive psychometric validation was not performed. Exploratory and confirmatory factor analyses, item-level psychometric assessment, and formal evaluation of measurement equivalence between the Arabic and English versions were not conducted. Therefore, the dimensional structure and cross-language equivalence of the instrument should be interpreted cautiously.

Moreover, although the questionnaire was informed by the TAM and selected UTAUT constructs, the study was not designed to evaluate these theoretical models via structural equation modeling or path analysis. Therefore, these frameworks serve as conceptual guides for questionnaire development rather than as formally tested theoretical models. Furthermore, the relatively lower reliability observed in the risk–reward appraisal scale (Cronbach’s α = 0.63) suggests potential measurement variability. Formal regression diagnostics, including calibration assessment, discrimination statistics, multicollinearity diagnostics, and influence analyses, were not included in the original statistical analysis. Consequently, the stability and calibration of the multivariable model cannot be fully assessed from the reported analyses. Because formal multicollinearity diagnostics were not performed, potential correlation among demographic characteristics and the attitudinal/perception domains cannot be completely excluded. Changes between unadjusted and adjusted estimates should therefore be interpreted as associations after statistical adjustment rather than evidence of causal relationships. Finally, the study did not assess actual clinical outcomes or verify the accuracy of the information obtained from ChatGPT; therefore, conclusions regarding its real-world impact on health decisions are limited. Future studies should perform comprehensive psychometric validation using exploratory and confirmatory factor analyses.

## 5 Conclusion

Among the adults recruited for this cross-sectional survey in the Western Region of Saudi Arabia, approximately two-thirds reported previous use of ChatGPT for health-related purposes. Use was more frequently reported among younger participants, those with higher educational attainment, and students, while more favorable perceptions were independently associated with reported use after adjustment. Participants generally recognized important limitations of ChatGPT and the continuing role of healthcare professionals in diagnosis and treatment decisions. However, these findings should be interpreted within the context of the nonprobability convenience sampling design and self-reported data and should not be considered a population prevalence estimate. Further representative and longitudinal studies are needed to determine the extent and consequences of health-related ChatGPT use across the wider Saudi population. Future research should explore the clinical implications of ChatGPT use, particularly its impact on health outcomes, patient–provider interactions, and healthcare utilization. Policymakers and healthcare providers should also consider strategies to responsibly integrate AI tools while minimizing the risks of misinformation and inappropriate self-diagnosis.

## Data Availability

The datasets presented in this article are not readily available because data supporting the findings of this study are available from the corresponding author upon reasonable request. Requests to access the datasets should be directed to almarghalani@tu.edu.sa.
