# Supplementary material for: Public knowledge, attitudes, and perceptions of the use of ChatGPT for self-diagnosis, health information, and medication use: a cross-sectional study
Source: Front Public Health. 2026 Oct 16;14:1936664. doi: 10.3389/fpubh.2026.1936664 (PMC13719601; doi:10.3389/fpubh.2026.1936664)
Supplement: Supplementary file 1 [file Data_Sheet_1.PDF]

Supplementary Table 1. STROBE Checklist for Cross-Sectional Studies "Public Knowledge, Attitude, and Perception of Using ChatGPT for Self-Diagnosis, Health Information, and Medication Use: A Cross-Sectional Study".

Section/Item	Item No.	Recommendation	Reported on Page(s)
**Title and Abstract**			
Title and abstract	1a	(a) Indicate the study's design with a commonly used term in the title or the abstract	Title; Abstract
	1b	(b) Provide in the abstract an informative and balanced summary of what was done and what was found	Abstract
Introduction			
Background/rationale	2	Explain the scientific background and rationale for the investigation being reported	Introduction
Objectives	3	State specific objectives, including any prespecified hypotheses	End of Introduction
Methods			
Study design	4	Present key elements of study design early in the paper	Section 2.1
Setting	5	Describe the setting, locations, and relevant dates, including periods of recruitment, exposure, follow-up, and data collection	Section 2.1
Participants	6	(a) Give the eligibility criteria, and the sources and methods of selection of participants	Section 2.2
Variables	7	Clearly define all outcomes, exposures, predictors, potential confounders, and effect modifiers. Give diagnostic criteria, if applicable	Section 2.7
Data sources/measurement	8	For each variable of interest, give sources of data and details of methods of assessment (measurement). Describe comparability of assessment methods if there is more than one group	Sections 2.4–2.7
Bias	9	Describe any efforts to address potential sources of bias	Sections 2.2 and Strengths & Limitations

Study size	10	Explain how the study size was arrived at	Section 2.3
Quantitative variables	11	Explain how quantitative variables were handled in the analyses. If applicable, describe which groupings were chosen and why	Sections 2.7–2.8
Statistical methods	12a	(a) Describe all statistical methods, including those used to control for confounding	Section 2.8
	12b	(b) Describe any methods used to examine subgroups and interactions	Not applicable
	12c	(c) Explain how missing data were addressed	Section 2.2 (Incomplete questionnaires excluded)
	12d	(d) If applicable, describe analytical methods taking account of sampling strategy	Section 2.2
	12e	(e) Describe any sensitivity analyses	Not applicable
Results			
Participants	13a	(a) Report numbers of individuals at each stage of study—e.g. numbers potentially eligible, examined for eligibility, confirmed eligible, included in the study, completing follow-up, and analyzed	Section 3.1
	13b	(b) Give reasons for non-participation at each stage	Incomplete questionnaires excluded
	13c	(c) Consider use of a flow diagram	Not applicable
Descriptive data	14a	(a) Give characteristics of study participants (e.g. demographic, clinical, social) and information on exposures and potential confounders	Table 1
	14b	(b) Indicate number of participants with missing data for each variable of interest	No missing data after exclusion
Outcome data	15	Report numbers of outcome events or summary measures	Tables 2–4; Figures 2–4
Main results	16a	(a) Give unadjusted estimates and, if applicable, confounder-adjusted estimates and their precision (eg, 95% confidence interval). Make clear which confounders were	Table 4

		adjusted for and why they were included	
	16b	(b) Report category boundaries when continuous variables were categorized	Tables 1 and 4
	16c	(c) If relevant, consider translating estimates of relative risk into absolute risk for a meaningful time period	Not applicable
Other analyses	17	Report other analyses done—eg analyses of subgroups and interactions, and sensitivity analyses	Sections 3.4–3.6
Discussion			
Key results	18	Summarise key results with reference to study objectives	Discussion
Limitations	19	Discuss limitations of the study, taking into account sources of potential bias or imprecision. Discuss both direction and magnitude of any potential bias	Strengths and Limitations
Interpretation	20	Give a cautious overall interpretation of results considering objectives, limitations, multiplicity of analyses, results from similar studies, and other relevant evidence	Discussion
Generalizability	21	Discuss the generalisability (external validity) of the study results	Strengths and Limitations; Conclusion
Other Information			
Funding	22	Give the source of funding and the role of the funders for the present study and, if applicable, for the original study on which the present article is based	Funding Statement

Supplementary Table 2. Intention to use ChatGPT for health-related purposes.

Item	Very unlikely %	Unlikely %	Neutral %	Likely %	Very Likely %	Mean ± SD
I am willing to use ChatGPT to self-diagnose health conditions.	15.2	30.5	24.4	21.2	8.7	2.78 ± 1.20
I am willing to use ChatGPT to seek information about medications.	7.8	13.9	19.2	45.0	14.1	3.44 ± 1.13
I will likely use ChatGPT as a primary source for health-related information in the future.	12.5	24.4	26.1	24.5	12.5	3.00 ± 1.22
I would recommend ChatGPT to others seeking health or medication information.	21.0	29.0	24.4	18.9	6.8	2.62 ± 1.20

Supplementary Table 3. Frequency of specific uses of ChatGPT for health-related purposes.

Item	Never %	Rarely %	Sometimes %	Often %	Always %
I have used ChatGPT to seek advice about beauty products.	12.7	14.3	16.2	31.7	25.2
I have used ChatGPT to seek advice about weight-loss drugs or supplements.	18.4	24.5	16.6	27.3	13.2
I have used ChatGPT to seek advice about acne medications.	17.1	25.3	20.7	22.3	14.6
I have used ChatGPT to seek advice about allergy medications.	21.6	29.9	19.5	18.2	10.7
I have used ChatGPT to seek advice about common cold remedies.	16.5	23.3	17.4	27.9	15.0
I have used ChatGPT to seek advice about sleep aids.	19.2	24.4	19.9	22.3	14.3
I have used ChatGPT to seek advice about contraceptives.	28.0	31.3	21.5	11.4	7.8
I have used ChatGPT to seek advice about pain relievers.	17.3	22.3	18.2	26.4	15.8

I have used ChatGPT to seek advice about antacids.	22.3	26.1	18.1	21.9	11.6
I have used ChatGPT to seek advice about vitamins or dietary supplements.	15.1	16.9	16.6	30.2	21.2
I have used ChatGPT to seek advice about diarrhea or constipation medications.	21.1	25.2	18.4	24.5	10.9
I have used ChatGPT to seek advice about antiemetic medications.	22.6	25.9	21.0	20.4	10.2
I have used ChatGPT to seek advice about medications used to reduce nicotine cravings during smoking cessation.	26.9	24.5	21.6	17.3	9.7
I have used ChatGPT to seek advice about medications used to numb localized pain from minor injuries, burns, or muscle soreness.	21.4	25.6	18.2	21.2	13.6
I have used ChatGPT to seek advice about medications used to reduce bloating or irregular bowel movements.	21.2	24.8	20.4	21.5	12.1

Supplementary Material S1

Survey Questionnaire

Public Knowledge, Attitude, and Perception of Using ChatGPT for Self-Diagnosis, Health Information, and Medication Use: A Cross-Sectional Study

Introduction

Dear Participant,

You are invited to participate in this anonymous survey investigating public knowledge, attitudes, and perceptions regarding the use of ChatGPT for health-related purposes. Participation is voluntary. Your responses will remain confidential and anonymous. The questionnaire takes approximately 5–7 minutes to complete.

By proceeding, you confirm that:

☐ **I am 18 years of age or older.**

☐ **I voluntarily agree to participate in this study.**

Section 1. Demographic Characteristics

1. Age

☐ **18–24 years**

☐ **25–44 years**

☐ **≥45 years**

---

2. Sex

☐ **Male**

☐ **Female**

---

3. Nationality

☐ **Saudi**

☐ **Non-Saudi**

---

4. Marital Status

☐ **Single**

☐ **Married**

☐ **Divorced/Widowed**

---

5. Educational Level

☐ **High school/Diploma or less**

☐ **Bachelor's degree**

☐ **Postgraduate degree (Master's/PhD)**

---

6. Employment Status

☐ **Student**

☐ **Employed**

☐ **Unemployed/Retired**

---

7. Do you have any chronic medical condition?

☐ **Yes**

☐ **No**

---

8. Smoking Status

☐ **Smoker**

☐ **Non-smoker**

---

9. How would you rate your health literacy?

☐ **Low**

☐ **Moderate**

☐ **High**

Section 2. ChatGPT Use

10. Have you ever used ChatGPT for health-related purposes?

☐ **Yes**

☐ **No**

Section 3. Knowledge

Please indicate your level of agreement with the following statements.

(1 = Strongly disagree, 2 = Disagree, 3 = Neutral, 4 = Agree, 5 = Strongly agree)

Statement	1	2	3	4	5
I understand that ChatGPT is not specifically designed for medical or health-related purposes.	○	○	○	○	○
I am aware that ChatGPT's health-related advice may not always be accurate or verified by medical professionals.	○	○	○	○	○
I know that using ChatGPT for self-diagnosis or medication advice could lead to incorrect health decisions.	○	○	○	○	○
I know that consulting a healthcare professional is necessary for an accurate diagnosis and safe medication use	○	○	○	○	○

Section 4. Attitudes

Statement	1	2	3	4	5
ChatGPT can provide helpful health information to support my decisions.	○	○	○	○	○
I feel confident using ChatGPT to learn about health conditions or medications without consulting a healthcare professional.	○	○	○	○	○
I think using ChatGPT for self-diagnosis is convenient because it is accessible anytime and anywhere.	○	○	○	○	○
I am worried that using ChatGPT for self-diagnosis or medication advice could lead to harmful health outcomes.	○	○	○	○	○
I believe ChatGPT is a trustworthy source for health information compared to other online sources	○	○	○	○	○

Section 5. Perceptions

Statement	1	2	3	4	5
ChatGPT can help me achieve my goals when seeking health information or medication advice.	○	○	○	○	○
Using ChatGPT reduces the effort needed to find information about health conditions or medications.	○	○	○	○	○
I have successfully obtained useful health or medication information from ChatGPT in the past.	○	○	○	○	○
I am satisfied with ChatGPT's ability to provide health-related information or medication advice.	○	○	○	○	○
ChatGPT helps me make informed and timely decisions about my health or medication use.	○	○	○	○	○
I am willing to make health-related decisions based on ChatGPT's recommendations.	○	○	○	○	○
ChatGPT provides clear and understandable recommendations for health and medication decisions.	○	○	○	○	○

Section 6. Risk–Reward Appraisal

Statement	1	2	3	4	5
The benefits of using ChatGPT for self-diagnosis or medication advice outweigh the potential risks.	○	○	○	○	○
I am worried that using ChatGPT for self-diagnosis or medication advice could lead to harmful health outcomes.	○	○	○	○	○
I trust ChatGPT to provide safe and reliable advice for nonprescription medications (OTC).	○	○	○	○	○
I believe the risks of using ChatGPT for health advice are lower than consulting unverified online sources.	○	○	○	○	○

Section 7. Intention to Use ChatGPT for Health-Related Applications

(1 = Very unlikely, 2 = Unlikely, 3 = Neutral, 4 = Likely, 5 = Very likely)

Statement	1	2	3	4	5
I am willing to use ChatGPT to self-diagnose health conditions.	○	○	○	○	○
I am willing to use ChatGPT to seek information about medications.	○	○	○	○	○
I will likely use ChatGPT as a primary source for health-related information in the future.	○	○	○	○	○
I would recommend ChatGPT to others seeking health or medication information.	○	○	○	○	○

Section 8. Specific Health-Related Uses of ChatGPT

How often have you used ChatGPT to seek advice about the following?

(1 = Never, 2 = Rarely, 3 = Sometimes, 4 = Often, 5 = Always)

Statement	1	2	3	4	5
I have used ChatGPT to seek advice about beauty products.	○	○	○	○	○
I have used ChatGPT to seek advice about weight-loss drugs or supplements.	○	○	○	○	○
I have used ChatGPT to seek advice about acne medications.	○	○	○	○	○
I have used ChatGPT to seek advice about allergy medications.	○	○	○	○	○
I have used ChatGPT to seek advice about common cold remedies.	○	○	○	○	○
I have used ChatGPT to seek advice about sleep aids.	○	○	○	○	○
I have used ChatGPT to seek advice about contraceptives.	○	○	○	○	○
I have used ChatGPT to seek advice about pain relievers.	○	○	○	○	○
I have used ChatGPT to seek advice about antacids.	○	○	○	○	○
I have used ChatGPT to seek advice about vitamins or dietary supplements.	○	○	○	○	○
I have used ChatGPT to seek advice about diarrhea or constipation medications.	○	○	○	○	○
I have used ChatGPT to seek advice about antiemetic medications.	○	○	○	○	○
I have used ChatGPT to seek advice about medications used to reduce nicotine cravings during smoking cessation.	○	○	○	○	○
I have used ChatGPT to seek advice about medications used to numb localized pain from minor injuries, burns, or muscle soreness.	○	○	○	○	○
I have used ChatGPT to seek advice about medications used to reduce bloating or irregular bowel movements.	○	○	○	○	○
